# Informal care for people with dementia in Europe

**DOI:** 10.1016/j.tjpad.2024.100015

**Published:** 2025-01-01

**Authors:** Ron Handels, Somboon Hataiyusuk, Anders Wimo, Anders Sköldunger, Christian Bakker, Anja Bieber, Alfonso Ciccone, Carlo Alberto Defanti, Andrea Fabbo, Sara Fascendini, Lutz Frölich, Chloé Gervès-Pinquié, Manuel Gonçalves-Pereira, Kate Irving, Raymond Koopmans, Patrizia Mecocci, Paola Merlo, Bernhard Michalowsky, Oliver Peters, Yolande Pijnenburg, Óscar Ribeiro, Geir Salbaek, Larissa Schwarzkopf, Hilde Verbeek, Marjolein de Vugt, Bob Woods, Orazio Zanetti, Bengt Winblad, Linus Jönsson, Actifcare consortium, ICTUS/DSA group, PLASA/DSA group, RECAGE consortium, RightTimePlaceCare consortium

**Affiliations:** a Alzheimer Centre Limburg, Faculty of Health Medicine and Life Sciences, Mental Health and Neuroscience Research Institute, Department of Psychiatry and Neuropsychology, Maastricht University, Universiteitssingel 40, 6200 MD, Maastricht, The Netherlands; b Division of Neurogeriatrics, Department of Neurobiology Care Sciences and Society; Karolinska Institutet; Sweden; BioClinicum J9:20, Akademiska stråket, 171 64 Solna, Sweden; c Department of Psychiatry, Faculty of Medicine Siriraj Hospital, Mahidol University, 2 Wang Lang Rd, 10700 Bangkok, Thailand; d Department of Primary and Community Care, Radboud university medical center, Geert Grooteplein Zuid 10, 6525 GA Nijmegen, the Netherlands; e Radboudumc Alzheimer Center, Geert Grooteplein Zuid 10, 6525 GA Nijmegen, the Netherlands; f Groenhuysen, Center for Geriatric Care, Bovendonk 29, 4707 ZH Roosendaal, the Netherlands; g Institute of Health and Nursing Sciences, Martin Luther University Halle-Wittenberg, 06108 Halle (Saale), Germany; h Department of Neurology with Neurosurgical Activity “Carlo Poma” Hospital, ASST di Mantova, Str. Lago Paiolo, 10, 46100 Mantova, MN, Italy; i Cognitive Disorders and Dementia Unit, Health Authority and Services (AUSL) of Modena, Strada Minutara Hangar 3, 41122 Modena, Italy; j FERB Alzheimer Centre, Ospedale Briolini, via A, Manzoni, 130, 24025 Gazzaniga, Italy; k Department of Geriatric Psychiatry, Central Institute of Mental Health, Medical Faculty Mannheim, University of Heidelberg, J 5 68159 Mannheim, Germany; l Health Economics & Outcomes Research (HEOR) unit, Real World Evidence (RWE) department, IQVIA, 17 bis Tsse, des Reflets, 92400 Courbevoie, France; m NOVA Medical School, Faculdade de Ciências Médicas, Universidade Nova de Lisboa; CHRC, REAL Associate Laboratory, Campo dos Mártires da Pátria 130, 1169-056 Lisboa, Portugal; n School of Nursing and Human Sciences, Dublin City University, Collins Ave Ext, Whitehall, Dublin, Ireland; o Joachim en Anna, center for specialized geriatric care, Groesbeekseweg 327, 6523 PA Nijmegen, the Netherlands; p Institute of Gerontology and Geriatrics, Department of Medicine and Surgery, Division of Clinical Geriatrics, University of Perugia, Piazza dell'Università 1, 06123 Perugia, PG, Italy; q Dept. of Neurology, Humanitas Gavazzeni, Via Mauro Gavazzeni 21, 24125 Bergamo, Italy; r German Center for Neurodegenerative Diseases (DZNE), Patient-reported Outcomes & Health Economics Research, Ellernholzstraße 1, 17489 Greifswald, Germany; s Charité-Universitätsme**diz**in Berlin, Campus Benjamin Franklin, Department of Psychiatry, Charitéplatz 1, 10117 Berlin, Germany; t Alzheimer Center Amsterdam, Neurology department, Vrije Universiteit Amsterdam, Amsterdam UMC, location VUmc, De Boelelaan 1118, 1081 HZ Amsterdam, The Netherlands; u Amsterdam Neuroscience, Neurodegeneration, De Boelelaan 1117, 1081 HV, Amsterdam, The Netherlands; v CINTESIS@RISE, Department of Education and Psychology, University of Aveiro – Campus, Universidade de Aveiro, 3810-193 Aveiro, Portugal; w Universitario de Santiago, Edf 5, 3810‑193 Aveiro, Portugal; x Norwegian National Advisory Unit on Ageing and Health, Vestfold Hospital Trust, Halfdan Wilhelmsens alle 17, 3103 Tønsberg, Norway; y Department of Geriatric Medicine, Oslo University Hospital, Sognsvannsveien 20, 0372, Oslo, Norway; z Institute of Clinical Medicine, Faculty of Medicine, University of Oslo, Problemveien 11, 0313 Oslo, Norway; aa IFT Institut für Therapieforschung, Mental Health and Addiction Research, Leopoldstrasse 175, 80804 Munich, Germany; ab Institute for Medical Information Processing, Biometry and Epidemiology, LMU Munich, Marchioninistrasse 17, 80336 Munich, Germany; ac Department of Health Services Research, Care and Public Health Research Institute, Faculty of Health Medicine and Life Sciences, Maastricht University, Duboisdomein 30, 6229 GT Maastricht, the Netherlands; ad Dementia Services Development Centre Wales, Bangor University, Bangor LL57 2DG, UK; ae IRCCS Istituto Centro San Giovanni di Dio Fatebenefratelli, Via Pilastroni, 4, 25125 Brescia, BS, Italy

**Keywords:** Dementia, informal care, health-economic evaluation, costs, resource use

## Abstract

**Introduction:**

Informal care estimates for use in health-economic models are lacking. We aimed to estimate the association between informal care time and dementia symptoms across Europe.

**Methods:**

A secondary analysis was performed on 13,529 observations in 5,369 persons from 9 European pooled cohort or trial studies in community-dwelling persons with dementia. A mixed regression model was fitted to time spent on instrumental or basic activities of daily living using disease severity and demographic characteristics.

**Results:**

Daily informal care time was 0.5 hours higher in moderate compared to mild and 1.3h higher in severe compared to mild cognitive impairment. Likewise, this was 1.2h and 2.7h for functional disability and 0.3h and 0.6h for behavioral symptoms in the same directions.

**Discussion:**

Estimates can be used in both single- and multi-domain health-economic models for dementia in European settings.

## 1 Background

With a global prevalence of 55 million individuals concerned [2] and a corresponding economic impact of US $1.3 trillion [3] dementia constitutes a substantial burden on societies worldwide. Core diagnostic aspects are cognitive decline interfering with everyday activities and behavioral and psychological symptoms [4]. Informal care forms one of the largest types of support [2]. In light of ageing societies with lower labor force the need for informal care is expected to increase.

Limited national care budgets force governments to make choices on how to spend their resources to provide persons with dementia and their informal caregivers with access to care and support. Health-economic simulation models support reimbursement decisions, especially to extrapolate evidence from trials with a limited follow-up period. Such models rely on estimates of (informal) care use and quality of life to simulate the natural course of dementia and the effect of an intervention [5].

Twelve recently (after 2010) published European-oriented models that included a reflection of informal care [[6], [7], [8]] varied widely in methods (such as an assumed amount of informal care costs, informal care time estimated by dependent or independent living, an interview-based estimation of informal care by disease severity states, or a regression-based estimation using data on cognitive impairment and/or functional disability and behavioral symptoms). For most studies, methodological details were lacking and for some they were only available in a local language or completely unavailable. In addition, model structures varied, reflecting cognitive impairment only or in combination with functional disability and behavioral symptoms, or based on full-time care. This hinders reproduction of estimates and re-use of estimates for future health-economic models. It leaves an urgent need to generate estimates of informal care time for dementia health-economic models serving a variety of model (multi-domain) structures and European settings [5].

We aimed to estimate the association between informal care time and dementia symptoms (cognitive impairment, functional disability and behavioral symptoms) adjusted for demographic characteristics across European settings using pooled data. We focus on informal caregiving time on instrumental and basic activities of daily living (ADL). We note that other types of impact of informal care [9] such as caregiver supervision time, productivity loss, physical and emotional burden, impact on health (depressive symptoms and comorbidities) and social life (loneliness), medical care and financial burden are outside the scope of our study.

The results of this study are intended as input estimates for dementia health-economic simulation models that represent disease progression by single or multiple (composite) domains.

## 2 Methods

A secondary analysis was performed on pooled data from studies involving informal care in dementia. Data were obtained from a variety of non-systematically identified studies. Studies were selected if: 1) the participants had a diagnosis of dementia at baseline established in a clinical setting (typically a memory clinic or general practitioner) and were living at home, 2) the study assessed informal care time using the (adjusted) Resource Use in Dementia (RUD) instrument, 3) the study assessed dementia symptoms in terms of global cognitive impairment, functional disabilities and/or behavioral symptoms, 4) the study provided information on basic demographic characteristics. We ad-hoc selected multi- and single-country longitudinal studies with a relatively large sample size. Included studies were Actifcare [10,11], DelpHi [1,12,13], ICTUS [14], IDA [[15], [16], [17]], NeedYD [18], PLASA [19], RECage [20], RTPC [21] and SQAD [22]. See Table 1 for study characteristics. The Swedish Ethical Review Authority provided ethical approval for conducting this study (Dnr 2022-01175-01). Each individual participating study had obtained ethics review for the original data collection and analysis. Data were pooled at a single location, and only a selection of the authors had access to the pooled data set.

**Table 1:** Characteristics of included studies.

	Actifcare	DelpHi	ICTUS	IDA	NeedYD	PLASA	RECage	RTPC	SQAD
Reference	[10,11]	[1,12,13]	[14]	[[15], [16], [17]]	[18]	[19]	[20]	[21]	[22]
Main aim	Understand why persons with dementia and their caregivers use or fail to use formal care services.	Test the efficacy and efficiency of implementing a support system for persons with dementia.	Describe the natural history, treatment outcomes and socioeconomic impact of individuals referred to dementia facilities in Europe.	Compare a complex intervention including caregiver support groups and counselling against usual care in terms of time to nursing home placement.	Investigate the (un)met needs of persons with early onset dementia and their family members.	Evaluate an AD care and assistance intervention in which community-dwelling persons with AD were evaluated bi-annually and in case of decline they received a specific intervention as well as information and training.	Evaluate the effectiveness of special care units for people with dementia and the behavioral and psychological symptoms of dementia.	Improve health services for European citizens with dementia by means of a longitudinal study with a 3 month follow-up.	Estimate the utilities and cost of medical care, community care and informal care.
Design	cohort	RCT	cohort	RCT	cohort	RCT	cohort	cohort	cohort
Sample size received data	451	634	1375	384	210	1131	382^1^	2014	272
Planned observation (months)	0, 6, 12	0, 12, 24	0, 6, 12, 18, 24	0, (6), 12, 24	0, 6, 12, 18, 24	0, (6), 12, (18), 24	0, 6, 12, 18, 24, 30, 36	0, 3	0, 6, 12
Recruitment settings	general practices, memory clinics, case managers, community mental health teams, newspapers	general practices	memory clinics	general practices	memory clinics, mental health services, specialized day care facilities	memory clinics in university hospitals and general hospitals	(memory) clinics	professional home care organizations and institutional long-term nursing care facilities	memory clinics
Countries	DE, IE, IT, NL, NO, PT, SE, UK	DE	BE, CH, DE, DK, ES, FR, GR, IT, NL, RO, SE, UK	DE	NL	FR	DE, IT	DE, EE, ES, FI, FR, NL, SE, UK	DK, FI, NO, SE
Cognition	MMSE	MMSE	MMSE	MMSE	MMSE	MMSE	MMSE	MMSE	MMSE
Instrumental ADL^2^	IADLS	B-ADL	IADLS	NOSGER	IDDD	IADLS	ADCS-ADL	n/a	n/a
Basic ADL^2^	PSMS	B-ADL	Katz	Barthel	IDDD	n/a	ADCS-ADL	Katz	n/a
Behavior	NPI-Q	NPI	NPI	n/a	NPI	NPI	NPI	NPI-Q	NPI-Q 10-item
Rating of ADL and behavior	Proxy	Proxy or self (in case informal caregiver was unavailable)	Proxy	Proxy	Proxy	Proxy	Proxy	Proxy	Proxy
Informal care	RUD	RUD	RUD	RUD	RUD	RUD	RUD	RUD	RUD

Country abbreviations: BE, Belgium; CH, Switzerland; DE, Germany; DK, Denmark; EE, Estonia; ES, Spain; FI, Finland; FR, France; GR, Greece; IE, Ireland; IT, Italy; NL, Netherlands; NO, Norway; PT, Portugal; RO, Romania; SE, Sweden; UK, United Kingdom.

Other abbreviations: RCT, randomized control trial; n/a = not available;

1 received a selection from countries Italy and Germany of project's full data (n=508).

2 proxy-rated in all studies, except in DelpHi study it was self-rated in case no informal caregiver was available.

### 2.1 Selection of participants and observations

**Figure 1:**
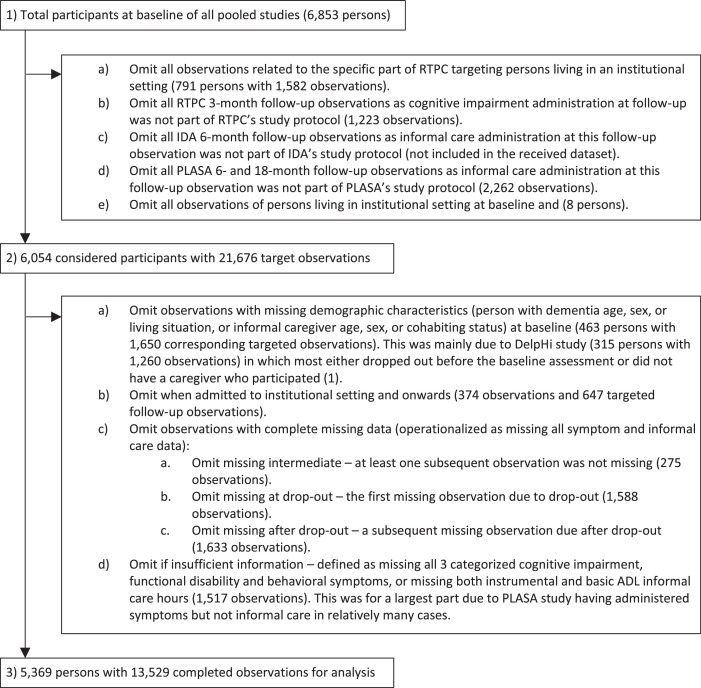
Selection of participants and observations from all pooled studies.

We omitted data from persons or observations with missing baseline demographics or missing cognitive or informal care data (case-wise deletion), either because these outcomes were not being part of the assessment protocol or because they were missing. Data during or after admission to an institutional setting were omitted (list-wise deletion) because generally studies lost them for follow-up, leaving those remaining likely to be a strong selection which could bias the results. Therefore, the institutional setting falls outside the focus of this study. See Fig. 1 for details.

### 2.2 Outcomes and harmonization

Informal care was assessed by the RUD (Resource Use in Dementia) instrument [23] or an adaptation of it, with data on the time spend providing basic ADL and the time spend providing instrumental ADL on a typical day, and the number of days in the past 30 days. The RUD instrument was originally developed in 1998 [24,25] and chosen for its wide used and validity, particularly in terms of the items on informal caregiving time [[26], [27], [28], [29], [30]]. Informal care hours on instrumental and basic ADL per day were summed and then truncated to 18 hours per day, assuming a minimum of 6 hours of sleep.

Demographic characteristics included age and sex of the person with dementia, and age, sex and cohabitation (i.e., whether the informal caregiver lives together with the person with dementia) of the informal caregiver.

Global cognitive impairment was reflected by the total score of the Mini-Mental State Examination (MMSE) [31], ranging from 0 (worse) to 30 (best).

Instrumental ADL were assessed by various instruments: Lawton's Instrumental Activities of Daily Living Scale (IADLS) [32], Bayer Activities of Daily Living Scale (B-ADL) [33], Nurses’ Observation Scale for Geriatric Patients (NOSGER) [34], Interview for Deterioration in Daily living Activities in Dementia (IDDD) [35], and Alzheimer's Disease Cooperative Study - Activities of Daily living (ADCS-ADL) [36]. The instrumental ADL assessment scales varied in terms of item topics and response options. In absence of availing mapping algorithms between all included ADL scales [37] we calculated the original scale's total score, then rescaled its minimum-maximum to 0-30 to map it to the Functional Activities Questionnaire (FAQ) total score [38]. This rescaled and mapped score was considered harmonized, under the assumption that the scales reflect the same concept of instrumental ADL, have similar floor/ceiling levels (i.e., their floor and ceiling reflect the same level abilities), and have a similar graduation or interval between response options.

Neuropsychiatric symptoms were assessed by the Neuropsychiatric Inventory Questionnaire (NPI) [39] on neuropsychiatric symptoms (delusions, hallucinations, agitation/aggression, depression/dysphoria, anxiety, elation/euphoria, apathy/indifference, disinhibition, irritability/lability, aberrant motor behavior, sleep and nighttime behavior disorders, and appetite and eating disorders). These behavioral and psychological symptoms are further shortened as behavioral symptoms.

Dementia Staging was assessed by the Clinical Dementia Rating (CDR) [40], characterizing six domains of cognitive and functional performance.

To allow our results to be used as input estimates for multi-domain health-economic models scales were transformed to a multi-domain disease-state descriptive system deployed by Green et al. [41] for cognitive impairment, functional disabilities and behavioral symptoms. Cognitive impairment was categorized as mild (MMSE total score higher or equal to 21), moderate [10–20], or severe (lower than 10). Functional disability in terms of instrumental ADL was categorized as no problems (0 to 8), moderately dependent [9–23], or highly dependent [24–30] based on the FAQ. Behavioral symptoms were categorized using the first 10 items of NPI(-Q/-NH) severity (omitting sleep/nighttime behaviors and appetite/eating items) as no or mild problems (all scored 1 or 0), moderate problems (at least one item is scored at 2 and all others less or equal than 2), or severe problems (at least one of the items is scored 3). Country was categorized into 5 European regions (Nordics, British Isles, Eastern Europe and Baltics, Southern Europe, Western Europe) as earlier defined [42].

## 3 Handling missing data

Missing data were handled in different ways. Minor missing data, such as missing a few scale items, were imputed by its mean or median. Missing demographics were carried forward from their last available observation. Drop-out was case-wise deleted. Cognitive impairment, functional disabilities, age of the person with dementia and cohabiting were significantly associated with drop-out, indicating the presence of selective drop-out. Partly missing data were conditional on observed data. Therefore, multiple imputation was performed to handle this type of missing data. See **supplementary 1** for details on missing data handling.

### 3.1 Statistical analysis

Baseline descriptive estimates per country and per region were generated.

For the base case analysis (i.e., primary analysis), a mixed model was fitted to the informal care hours with a random intercept reflecting observations nested within individuals and individuals nested within the studies. Independent variables were the person with dementia age and sex, informal caregiver age, sex and living together, cognitive impairment, functional disabilities and behavioral symptoms (all 3 categorized), and region. With observations nested within an individual, we tempted the coefficients to reflect the change in informal care hours associated with a change in the independent variables.

Predicted values were generated to act as input estimates for health-economic models. These were generated by symptom, person with dementia age and region (at mean estimate of the other factors per region).

Post-hoc, various additional analyses were performed to serve alternative health-economic model structures that reflected only cognitive impairment, reflected a composite of cognitive impairment and functional disabilities, or reflected these outcomes on a continuous instead of categorized level. In addition, sensitivity analyses were performed to address the sensitivity of the results to methodological choices and to support the interpretation of the results.

A spreadsheet tool was developed to predict mean informal care hours by dementia severity per region to support health-economic modelers to obtain input estimates that fit their specific health-economic model structure.

## 4 Results

We selected a total of 5,369 persons with 13,529 completed observations from the pooled studies for analysis (see Fig. 1). Participants at baseline had a mean age of 79 (SD=8) and 63 % were female, with 3.3 hours per day of received informal care ranging from 1.1 to 7.5 between countries. Informal caregivers had a mean age of 63 (SD=14), 67 % were female and 64 % cohabited with the person with dementia. See Table 2 for details.

**Table 2:** Baseline characteristics by country.

country	BE	CH	DE	DK	EE	ES	FI	FR	GR	IE	IT	NL	NO	PT	RO	SE	UK	Total
n	55	32	913	80	171	407	227	1534	113	42	449	519	105	66	70	349	237	5369
Age, years (PwD)	74 (7)	77 (7)	80 (7)	77 (8)	82 (8)	80 (7)	81 (7)	80 (6)	73 (7)	74 (9)	79 (7)	71 (11)	79 (6)	78 (6)	76 (9)	78 (8)	80 (7)	79 (8)
Female sex (PwD)	60 %	72 %	61 %	58 %	74 %	68 %	65 %	69 %	60 %	52 %	62 %	53 %	60 %	62 %	54 %	54 %	54 %	63 %
Cognitive impairment																		
Mild	62 %	50 %	44 %	58 %	6 %	32 %	32 %	45 %	62 %	51 %	33 %	42 %	46 %	26 %	50 %	46 %	45 %	41 %
Moderate	37 %	50 %	49 %	39 %	53 %	62 %	57 %	53 %	38 %	42 %	60 %	47 %	51 %	68 %	50 %	44 %	45 %	51 %
Severe	1 %	0 %	8 %	4 %	41 %	6 %	12 %	2 %	0 %	7 %	8 %	12 %	3 %	6 %	0 %	10 %	10 %	7 %
Functional disability																		
Mild	53 %	59 %	25 %	36 %	4 %	19 %	17 %	29 %	52 %	21 %	14 %	18 %	21 %	18 %	31 %	22 %	15 %	24 %
Moderate	40 %	22 %	53 %	51 %	35 %	53 %	54 %	56 %	36 %	43 %	65 %	53 %	63 %	61 %	50 %	53 %	56 %	54 %
Severe	7 %	19 %	22 %	13 %	61 %	28 %	29 %	15 %	12 %	36 %	21 %	29 %	16 %	21 %	19 %	25 %	28 %	23 %
Behavioral symptoms																		
Mild	31 %	22 %	36 %	49 %	16 %	28 %	33 %	32 %	31 %	14 %	10 %	28 %	23 %	27 %	20 %	45 %	34 %	30 %
Moderate	42 %	72 %	32 %	43 %	36 %	36 %	39 %	37 %	27 %	52 %	27 %	29 %	49 %	47 %	71 %	24 %	32 %	35 %
Severe	27 %	6 %	32 %	9 %	48 %	36 %	28 %	31 %	42 %	33 %	63 %	43 %	29 %	26 %	9 %	30 %	33 %	35 %
Informal care (hour/day)	1.1 (1.8)	1.7 (1.5)	3.8 (3.5)	1.9 (3.0)	7.5 (6.0)	4.4 (4.7)	3.5 (4.1)	2.3 (2.9)	2.5 (4.0)	2.4 (2.8)	4.3 (3.8)	3.1 (3.0)	2.8 (2.7)	2.4 (1.6)	4.5 (4.8)	2.9 (3.3)	4.8 (4.9)	3.3 (3.8)
Age, years (IC)	62 (13)	63 (14)	62 (13)	66 (13)	57 (13)	65 (14)	64 (13)	64 (14)	56 (15)	58 (15)	60 (13)	63 (12)	67 (14)	65 (15)	62 (13)	68 (13)	68 (13)	63 (14)
Female sex (IC)	69 %	59 %	73 %	61 %	76 %	67 %	58 %	64 %	66 %	76 %	71 %	61 %	66 %	67 %	70 %	64 %	68 %	67 %
Cohabit (IC with PwD)	58 %	50 %	63 %	59 %	70 %	81 %	49 %	57 %	81 %	57 %	55 %	77 %	65 %	85 %	74 %	65 %	73 %	64 %

Country abbreviations: BE, Belgium; CH, Switzerland; DE, Germany; DK, Denmark; EE, Estonia; ES, Spain; FI, Finland; FR, France; GR, Greece; IE, Ireland; IT, Italy; NL, Netherlands; NO, Norway; PT, Portugal; RO, Romania; SE, Sweden; UK, United Kingdom

The results of the base case statistical analysis are presented in Table 3 and indicated that sex, cognitive impairment, functional disability and behavioral symptoms of the person with dementia, and age, sex and cohabiting of the informal caregiver were significantly associated with informal care time. Informal care time was lower for female persons with dementia and increased with cognitive impairment, functional disability and behavioral symptoms, with its effect size being largest for functional status. Informal care time ranged almost 3 hours per day across regions, being highest in Eastern Europe & Baltic and lowest in Nordic countries.

**Table 3:** Results of the base case statistical analysis and additional analyses (beta coefficients and 95 % confidence interval from mixed linear regression with dependent variable hours of informal care per day on instrumental and basic activities of daily living).

	3-domain categorized^3^ (base case)	MMSE categorized^3^	MMSE sum^4^	3-domain continuous^4^	CDR-global^5^	CDR sum of boxes categorized^3^^,^^5^	CDR sum of boxes^5^	MMSE categorized^3^ additional data^5^
n	13,529	13,529	12,330	8,680	4,227	4,399	4,673	14,006
Cognitive impairment (MMSE) or CDR	⁎⁎	⁎⁎	-0.16 (-0.17 to -0.15)⁎⁎	-0.05 (-0.07 to -0.04)⁎⁎	⁎⁎	⁎⁎	0.39 (0.36 to 0.41)⁎⁎	⁎⁎
Moderate^1^	0.51 (0.37 to 0.64)	1.00 (0.87 to 1.12)			1.40 (1.19 to 1.62)	1.68 (1.46 to 1.90)		0.96 (0.83 to 1.08)
Severe^1^	1.29 (1.04 to 1.54)	2.36 (2.13 to 2.59)			4.12 (3.64 to 4.61)	4.14 (3.60 to 4.67)		2.26 (2.02 to 2.49)
Functional disability (mapped FAQ)	⁎⁎			0.14 (0.12 to 0.15)⁎⁎				
Moderate^1^	1.14 (0.99 to 1.29)							
Severe^1^	2.65 (2.43 to 2.87)							
Behavior symptoms (NPI-Q)	⁎⁎			0.06 (0.05 to 0.07)⁎⁎				
Moderate^1^	0.34 (0.20 to 0.48)							
Severe^1^	0.61 (0.45 to 0.77)							
Age, years (PwD)	-0.01 (-0.02 to 0.00)	0.02 (0.00 to 0.03)*	0.02 (0.00 to 0.03)*	-0.02 (-0.03 to 0.00)⁎⁎	0.01 (-0.01 to 0.03)	0.01 (-0.01 to 0.04)	0.01 (-0.01 to 0.03)	0.01 (0.00 to 0.02)
Female sex (PwD)	-0.35 (-0.55 to -0.15)⁎⁎	-0.50 (-0.71 to -0.29)⁎⁎	-0.55 (-0.76 to -0.33)⁎⁎	-0.34 (-0.58 to -0.09)⁎⁎	-0.02 (-0.42 to 0.37)	0.00 (-0.39 to 0.38)	-0.11 (-0.45 to 0.22)	-1.30 (-1.48 to -1.13)⁎⁎
Cohabit (IC with PwD)	1.41 (1.25 to 1.58)⁎⁎	1.50 (1.33 to 1.67)⁎⁎	1.42 (1.24 to 1.61)⁎⁎	1.04 (0.83 to 1.26)⁎⁎	1.36 (1.01 to 1.71)⁎⁎	1.33 (0.99 to 1.67)⁎⁎	1.02 (0.77 to 1.26)⁎⁎	
Age, years (IC)	0.03 (0.02 to 0.04)⁎⁎	0.03 (0.02 to 0.04)⁎⁎	0.03 (0.02 to 0.04)⁎⁎	0.03 (0.02 to 0.04)⁎⁎	0.03 (0.01 to 0.04)**	0.03 (0.01 to 0.04)⁎⁎	0.03 (0.02 to 0.04)⁎⁎	
Female sex (IC)	0.41 (0.21 to 0.60)⁎⁎	0.48 (0.28 to 0.69)⁎⁎	0.51 (0.31 to 0.72)⁎⁎	0.33 (0.09 to 0.57)⁎⁎	0.62 (0.23 to 1.01)⁎⁎	0.61 (0.22 to 1.00)⁎⁎⁎	0.52 (0.18 to 0.86)⁎⁎	
Region	⁎⁎	⁎⁎	⁎⁎	⁎⁎	⁎⁎	⁎⁎	⁎⁎	⁎⁎
British Isles^2^	1.55 (1.13 to 1.97)	1.71 (1.26 to 2.17)	1.38 (0.92 to 1.84)	0.89 (0.36 to 1.42)	1.02 (0.39 to 1.64)	1.10 (0.48 to 1.72)	1.14 (0.58 to 1.70)	1.91 (1.44 to 2.39)
Eastern Europe and Baltics^2^	2.84 (2.37 to 3.32)	3.08 (2.58 to 3.57)	2.85 (2.36 to 3.35)	2.33 (1.62 to 3.04)	3.73 (2.74 to 4.73)	3.54 (2.59 to 4.49)	2.66 (1.91 to 3.41)	3.17 (2.66 to 3.69)
Southern Europe^2^	1.48 (1.15 to 1.80)	1.72 (1.37 to 2.08)	1.50 (1.15 to 1.86)	1.03 (0.60 to 1.47)	1.64 (1.12 to 2.17)	1.61 (1.10 to 2.13)	1.61 (1.16 to 2.07)	1.86 (1.49 to 2.23)
Western Europe^2^	0.17 (-0.13 to 0.48)	0.20 (-0.13 to 0.53)	0.01 (-0.33 to 0.34)	0.04 (-0.39 to 0.48)	-0.27 (-0.78 to 0.25)	-0.28 (-0.78 to 0.23)	-0.17 (-0.63 to 0.28)	0.25 (-0.10 to 0.60)
Constant	-1.13 (-2.11 to -0.14)	-1.84 (-2.92 to -0.76)	1.96 (0.86 to 3.06)	0.12 (-1.09 to 1.32)	-1.63 (-3.37 to 0.11)	-1.81 (-3.50 to -0.11)	-3.76 (-5.22 to -2.30)	1.62 (0.23 to 3.01)

⁎ p<0.05,

⁎⁎ p<0.01

1 Reference category is mild.

2 Reference is Nordics.

3 MSME mild (21-30), moderate (10-20), severe (0-10); FAQ mild (0-8), moderate (9-23), severe (24-30); NPI(-Q) first 10 severity items mild (all scored 1 or 0), moderate (at least one item is scored at 2 and all others less or equal than 2), severe (at least one of the items is scored 3); CDR sum of boxes mild (4.5-9), moderate (9.5-15.5), severe (16-18)

4 Per point improvement on MMSE sum score (0-30), per point worsening on mapped FAQ score (0-30), per point worsening on NPI-Q score (0-36).

4 Based on data from Actifcare and ICTUS studies only.

5 Additional data from Gervès-Pinquié et al.(44) and Pires et al.(45). From Pires et al. the Global Deterioration Scale (GDS) was used as MMSE was not available. Abbreviations: CDR, Clinical Dementia Rating; FAQ, Functional Activities Questionnaire; IC, informal caregiver; MMSE, Mini-Mental State Examination; NPI, Neuropsychiatric Inventory; PwD, person with dementia.

Informal care time was 0.5 hours per day higher in moderate compared to mild and 1.3 higher in severe compared to mild cognitive impairment (resulting in 0.8 higher in severe compared to moderate). Likewise, informal care time was 1.2 higher in moderate and 2.7 higher in severe, compared to mild functional disability, and 0.3 higher in moderate and 0.6 higher in severe compared to mild behavioral symptoms; adjusted for other factors. See **supplementary material 2** for a spreadsheet tool to predict mean informal care hours by dementia severity per region for single or combined factors.

Regression diagnostics indicated residuals were not normally distributed (see **Figure S3.1** and **Table S3.1** for regression details). Mean predicted values per decile of predicted values mostly fell outside the 95 % confidence intervals of observed mean informal care hours corresponding to those deciles (see **figure S3.2**). Observed informal care time stratified by symptoms ranged from about 1 to 7 hours per day. Compared to predicted informal care hours deviation was up to about 1 hour. We judged this as a moderate to good fit. See **Figure S3.3** for details.

### 4.1 Additional analyses

The results of the additional analyses are presented in Table 3 and **supplementary material 4**.

First, a model using MMSE categorized adjusted for demographics estimated an informal care increase of 1.0 hours from mild to moderate cognitive impairment and 2.4 hours from mild to severe.

Second, a model using MMSE total score (range 0-30; non-imputed) adjusted for demographics estimated an informal care increase of 0.16 hours per point MMSE drop.

Third, a model using cognitive impairment, functional disability and behavioral symptoms as continuous outcomes adjusted for demographics estimated an informal care increase of 0.05 hours per point drop MMSE total score (range 0-30), 0.14 hours per point mapped FAQ (range 0-30) and 0.06 hours per point NPI-Q total (range 0-36).

Fourth, a model using CDR global score (non-imputed) adjusted for demographics estimated an informal care increase of 1.4 hours from mild to moderate and 4.1 hours from mild to severe dementia, in sub-selection of datasets Actifcare and ICTUS with CDR-global score available.

Fifth, a model using CDR sum of boxes categorized (mild: 4.5-9, moderate: 9.5-15.5, severe: 16-18 [43]) adjusted for demographics estimated an informal care increase of 1.7 hours from mild to moderate and 4.1 hours from mild to severe dementia.

Sixth, a model using CDR sum of boxes (range 0-18; non-imputed) adjusted for demographics estimated an informal care increase of 0.39 hours per point on the CDR sum of boxes score.

Seventh, additional data were obtained from Gervès-Pinquié et al. [44] and Pires et al. [45] with cross-sectional and limited information on clinical symptoms and demographic characteristics of informal caregivers. A model was fit to MMSE categorized, or if missing global deterioration scale, and adjusted for person with dementia age and sex only. Informal care increased by 1.0 hours from mild to moderate and by 2.3 hours from mild to severe cognitive impairment.

All additional analyses were added to the spreadsheet tool to predict mean informal care hours per region for single or combined factors (see **supplementary material 2**).

### 4.2 Sensitivity analyses

First, hours of instrumental and basic ADL were summed before imputation. The same factors were significant compared to the base case and coefficients differed by 0.1 hours or less. In addition, the base case model was applied separately to instrumental ADL and basic ADL (excluding SQAD as informal care was not administered separately). Compared to the base case, the sex of the person with dementia and the age of the informal caregiver were no longer significant for personal ADL, and the age of the person with dementia was significant for instrumental ADL. Coefficients differed by 0.3 hours or less. See **Figure S5.1** for details.

Second, the base case and additional analyses for ‘categorized MMSE only’, ‘CDR global’ and ‘CDR sum of boxes categorized’ were performed on the same sub-sample of those with all outcomes available (i.e., Actifcare and ICTUS) to assess the sensitivity to the outcome scale used. Severe compared to mild cognitive impairment was associated with 1.7 hours of informal care when adjusted for functional disability and behavioral symptoms (base case model), with 2.8 hours when not adjusted (categorized MMSE only analysis), with 4.2 hours when CDR composite outcome of cognitive impairment and functional disability was used (CDR-global analysis) and with 4.1 hours for CDR sum of boxes (CDR sum of boxes categorized analysis). See **figure S5.1** for details.

Third, study-specific results indicated variation in the magnitude between dementia symptoms and informal care hours. The effect of functional disability differed significantly between studies (statistically tested with an interaction between study and cognitive impairment (p=0.055), functional disability (p<0.001) and behavioral symptoms (p=0.326)). For example, studies Actifcare, ICTUS, IDA, PLASA, RTPC and SQAD showed about twice as high association between severe functional disability and informal care hours than the other studies (DelpHi, NeedYd and RECage). See **Figure S5.2** for details.

Fourth, interactions between clinical symptoms and all covariates were tested and found significant for caregiver age (the effect of functional disability and behavioral symptoms on informal care was about 0.01 to 0.02 hour higher with each year of increasing age), cohabit (the effect of cognitive impairment, functional disability and behavioral symptoms on informal care was about 0.4 to 1.3 hour higher when cohabiting) and region (the effect of functional disability and behavioral symptoms on informal care were up to 1 hour different between regions with one exception of 2.8 hours different effect).

## 5 Discussion

Informal care time was estimated in pooled longitudinal data from 5,369 persons with mild, moderate or severe dementia from various European countries. Informal care time increased by 0.5 hours per day between mild and moderate cognitive impairment and by 1.3 between mild and severe cognitive impairment, and by 1.2 (mild-moderate) and 2.7 (mild-severe) for functional disability and 0.3 (mild-moderate) and 0.6 (mild-severe) for behavioral symptoms respectively. A prediction tool was developed to generate health-economic model input estimates.

The results can be interpreted in various ways. Functional disability was most substantially associated to informal care time, which could imply functional disability to be an important driver of the consumed care by persons with dementia. However, functional disability was expected to be the strongest predictor because we operationally defined informal care time from the questions on the RUD instrument on hours of support on instrumental and basic activities of daily living.

The combination of cognitive impairment and functional disability (measured by the composite measure CDR-global) was related to 4.1 hours of informal care, which was similar to the combination of severe cognitive impairment and severe functional disability (1.3+2.7). This implies a consistency in our results, making the choice of measure in a health-economic model (single composite or combination of separate outcomes) unlikely to drive health-economic modeling results.

### 5.1 Comparison to literature

Our results align with the findings from systematic reviews that informal care time increases with disease severity in terms of cognitive impairment and/or functional disability [9,42,[46], [47], [48]]. Angeles et al. [46] and Wolfs et al. [49] also indicated behavioral symptoms as a driver of caregiving time. Our study adds specific estimates of informal care time readily available for use in health-economic models.

Two large multi-country studies showed an increase of 1.0 and 2.6 hours per day from moderate and severe compared to mild MMSE [50] and 1.3 and 2.4 respectively [51], which seemed comparable to our most alike MMSE-only analysis result of 1.0 and 2.4 respectively. Unfortunately, we were not able to include the data of about 5000 individuals from these two and one additional [52] large multi-country studies (due to difficulty reaching the data owner or difficulty to pool the data due to virtual access only). However, the similarly observed estimates support the validity of our results and imply little impact of missing out these data.

In a previous meta-analysis, caregiving time was ranked highest to lowest in British Isles, Southern Europe, Western Europe, Nordics and Eastern Europe and Baltics [42], which differed from our results placing Eastern Europe and Baltics highest. This could be due to small sample size as only two studies were included in the meta-analysis on this region and only two countries reflected this region in our study. Other reviews referred to no [9] or some difference between countries such as Italy relying more and Sweden relying less on informal care due to differences in planned social care distribution [48]. However, they are limited in their comparison among a relatively small set of countries and it remains uncertain whether country or study method was the cause for such differences.

### 5.2 Recommendations

The results of this study can be used as input estimates for health-economic simulation models in dementia diseases that represent disease progression by multiple domains [[53], [54], [55]] as well as models that represent single or composite domains. We note the need to use the same (categorized) scale in a model to enable implementation of our results.

Models reflecting disease progression by a single domain run the risk of confounding (e.g., informal care seems driven by cognitive impairment while in fact part of the informal care is driven by functional disability, which is correlated to cognitive impairment) when evaluating an intervention. This was supported by our sensitivity analysis showing a relatively larger effect of cognitive impairment when no longer adjusted for functional disability and behavioral symptoms. This was further supported by the effect of CDR matching the effect of cognitive impairment and functional disability combined. Therefore, if the impact of an intervention is reflected by a health-economic simulation model to only affect a single domain such as cognitive impairment (e.g., by cognitive stimulation therapy with consistent effects on cognition and inconsistent effect on functioning and behavioral symptoms [56]) we recommend using adjusted estimates from our base case analyses. Using estimates from our additional analysis on cognition-only or CDR in this situation would run the risk of allocating benefits on informal care that are driven by functional disability and behavioral symptoms that are normally correlated but unaffected by an intervention only affecting a specific domain. If, however, an intervention affects all domains simultaneously (i.e., slowing progression) we recommend adding the hours related to cognitive impairment, functional disability and behavioral symptoms (proportional to their occurrence across natural disease progression) or use the estimates from the CDR composite outcome.

We recommend using our spreadsheet tool (see **supplementary material 2**) to predict informal care hours picking the base case or additional analyses that matches the design of the health-economic simulation model and nature of the intervention. There are various proposed methods for valuing informal care time into health or monetary terms [57] but these fell outside the scope of this study.

At last, we note the confidence intervals reported in this study do not represent methodological uncertainty, which was partly reflected by the variation from our sensitivity analyses (see strengths and limitations) and partly unaddressed such as uncertainty of assumptions on mapping and handling missing data due to drop-out. We recommend reflecting this additional uncertainty in a health-economic simulation model uncertainty analysis.

## 6 Strengths and limitations

The significant and relatively large variation between studies limits the robustness of the results. The variation could be explained by various reasons, for example the focus of the NeedYd study on young-onset dementia, where informal care is likely to be provided by a working spouse, compared with other studies where the cohabiting spouse is often retired, as well as the specific setting of (specialized) day care. Also, Actifcare and RTPC studies targeted a population of persons with dementia likely in need of additional formal care, possibly reflecting more burdened informal caregivers. These differences between studies have probably also caused the different outcomes observed in the second sensitivity analysis in which a selection of data from specific studies was used. This variation supports the value of pooled data analysis providing an average estimate across studies. The relatively small variation related to the methodological choice of summing informal care before imputation or assessing informal care on instrumental ADL and basic ADL separately support the robustness of the results.

Regression assumptions were violated or not tested due to difficulties in obtaining them from a mixed model fitted to multiple imputed data. Although mean predicted values deviated from mean observed values, we judge their deviation (less than 1 hour per day) as moderate to small compared to the range of about 1-7 hours of informal care per day.

Generalization is limited for various reasons. We did not adjust for drop-out, possibly underestimating informal care time in case of drop-out due to overburdened caregivers. On the other hand, informal caregiving time is likely overestimated as those providing consent for participation have been shown to have higher time investment and caregiver burden [58]. Results are generalizable to the various inclusion criteria and settings of each study, their convenient sampling method, the underrepresentation of Eastern European countries, informal care time related to instrumental and basic activities of daily living (not supervision) and community living (as institutional setting fell outside our scope). A relatively low number of individuals were living at home and identified with severe cognitive impairment (6 %) and severe functional disability (10 %), which we did not test for its difference to natural prevalence and possibly reflect a selection bias (other than omitting due to institutionalization). We did not adjust for comorbidities, which has not been studied widely [42,48] but could potentially be a driver of informal care unrelated to dementia. Only the primary caregiver hours were included, not reflecting the contribution of multiple informal caregivers for a single person with dementia [59].

## 7 Conclusions

Informal care time increased with increasing severity in cognitive impairment, functional disability and behavioral symptoms, adjusted for demographic characteristics in pooled longitudinal data from 5,369 persons with dementia from various European countries. Estimates can be used in single or multi-domain health-economic simulation models related to dementia.

## Precis

Estimate of informal care time by dementia symptoms in a pooled dataset of more than 5000 individuals with dementia for use in health-economic models.

## Author contributions

Concept and design: RH, LJ

Acquisition of data: MdV, AB, IK, SG, BW, GPM, OZ, AW, BM, CGP, LS, YP, RK, CB, AD, SF, AC, AF, LF, PM, PM, OP, OR, HV, LJ

Analysis and interpretation of data: RH, SH (as part of a thesis for a Master of Science internship supervised by RH), LJ

Statistical analysis: RH

Drafting the manuscript: RH, SH

Critical revision: all

## Details on author study group or consortium

**ICTUS/DSA Group** refers to: ICTUS study Group: Vellas B., Reynish E., Ousset PJ., Andrieu S. (Toulouse), Burns A. (Manchester),Pasquier F. (Lille), Frisoni G.(Brescia),Salmon E. (Liège), Michel J.P., Zekry D.S. (Geneva), Boada M. (Barcelona), Dartigues J.F. (Bordeaux), Olde-Rikkert M.G.M. (Nijmejen), Rigaud A.S. (Paris), Winblad B. (Huddinge), Malick A., Sinclair A. (Warwick), Frölich L.(Mannheim), Scheltens P. (Amsterdam), Ribera C.(Madrid), Touchon J. (Montpellier), Robert P. (Nice), Salva A.(Barcelona), Waldmar G.(Copenhagen),Bullock R.(Swindon), Costa-Tsolaki M. (Thesaloniki), Rodriguez G. (Genoa), Spiru L. (Bucharest), Jones R.W. (Bath), Stiens G., Stoppe G. (Goettingen), Eriksdotter Jönhagen M. (Stockholm), Cherubini A. (Perugia), Lage P.M., Gomez-Isla T. (Pamplona), Camus V. (Tours), Agüera-Morales E., Lopez F.(Cordoba). DSA Group: Andrieu S., Savy S., Cantet C., Coley N.

**PLASA/DSA Group** refers to: PLASA study Group: Vellas B., Nourhashemi F., Gillette-Guyonnet S., Andrieu S. (Toulouse University Hospital UH); Quinçon A. (Albi General Hospital GH); Peju L. (Ales GH); Berrut G. (Anger UH); Picot F. (Annecy GH); De Guio G. (Bar Le Duc GH); Rainfray M. (Bordeaux UH); Gentric A. (Brest UH); Tannier C. (Carcassonne GH); Taillez N. (Carvin GH); Declippeleir F., Hohn C. (Chambéry GH); Maugourd M.F. (Champcueil GH); Pesque T. (Dieppe GH); Simon T. (Elbeuf GH); Ribiere J. (Grasse GH); Franco A., Couturier P. (Grenoble UH); Bordes S. (Lannemezan GH); De Pemille F. (Lavaur GH); Landrin I. (Le Havre GH); Senechal O. (Lens GH); Roche J., Pasquier F. (Lille UH); Bonnefoy M. (Lyon UH); Michel B. (Marseille UH); Jeandel C., Touchon J. (Montpellier UH); Giordana J.Y. (Nice GH); Brocker P., Robert P., Guerin O. (Nice UH); Strubel D. (Nimes GH); Chaumier J.A. (Niort GH); Durand-Gasselin B. (Paris GH); Samson M. (Paris GH); Belmin J., Legrain S., Rigaud A.S., Teillet L., Verny M., Pariel S. (Paris UH); de la Fournière F. (Pau GH); Drunat O. (Plaisir GH); Blanchard F. (Reims UH); Jouanny P. (Rennes UH); Forzy P. (Roubaix GH); Moynot Y. (Rouen GH); Chassagne P., Hannequin D., Dugny F., Levasseur C. (Rouen UH); Aubertin A. (Saint Dizier GH); Quignard E. (Sezanne GH); Leurs P. (Valenciennes GH); Le Provost C. (Vannes GH); Feteanu D., Trivalle C. (Villejuif GH); Frigard B. (Wasquehal GH). DSA Group: Andrieu S., Cantet C., Coley N.

**Actifcare Consortium**: The Actifcare Consortium partners are: Coordinator: Maastricht University (NL): Frans Verhey, professor (scientific coordinator, WP1 leader) Consortium members: Maastricht University (NL): Marjolein de Vugt, Claire Wolfs, Ron Handels, Liselot Kerpershoek. Martin-Luther University Halle Wittenberg (DE): Gabriele Meyer (WP2 leader), Astrid Stephan, Anja Bieber, Anja Broda, Gabriele Bartoszek. Bangor University (UK): Bob Woods (WP3 leader), Hannah Jelley. Nottingham University (UK): Martin Orrell, Karolinska Institutet (SE): Anders Wimo (WP4 leader), Anders Sköldunger, Britt-Marie Sj:olund, Oslo University Hospital (NW): Knut Engedal, Geir Selbaek (WP5 leader), Mona Michelet, Janne Rosvik, Siren Eriksen. Dublin City University (IE): Kate Irving (WP6 leader), Louise Hopper, Rachael Joyce. Nova Medical School, Faculdade de Ciências Médicas, Universidade Nova de Lisboa (PT): Manuel Gonçalves-Pereira, Maria J. Marques, M. Conceição Balsinha, Ana Machado, on behalf of the Portuguese Actifcare team. Alzheimer's Research Unit-Memory Clinic, IRCCS “Centro S.Giovanni di Dio (IT): Orazio Zanetti, Daniel Portolani

**RECAGE consortium**: C.A. Defanti, Fondazione Europea di Ricerca Biomedica (FERB Onlus), Gazzaniga, Italy; S. Bergh, Innlandet Hospital Trust, The Research center for Age-related Functional Decline and Disease, Ottestad, Norway; G. Frisoni, Division of Geriatrics, Department of Rehabilitation and Geriatrics, Geneva University Hospital and Geneva University, Switzerland; A. Fabbo, Azienda Unità sanitaria locale di Modena, Primary Care Department- Dementia Programme, Modena, Italy; M. Tsolaki, Greek Association of Alzheimer's Disease and Related Disorders (GAADRD), Thessaloniki, Macedonia, Hellas; L. Froelich, Zentralinstitut Fuer Seelische Gesundheit, Department of Geriatric Psychiatry, Mannheim, Germany; L. Hausner, Zentralinstitut Fuer Seelische Gesundheit, Department of Geriatric Psychiatry, Mannheim, Germany; A. Ciccone, Dipartimento di Neuroscienze ASST di Mantova, Italy; O. Peters, Department of Psychiatry and Psychotherapy Charité Universitätsmedizin Berlin, Germany; P. Merlo, Humanitas Gavazzeni, Department of Neurology, Bergamo, Italy; P. Mecocci and A. Giulia Guazzarini, Institute of Gerontology and Geriatrics, Department of Medicine and Surgery, University of Perugia, Italy and Division of Clinical Geriatrics, NVS Department, Karolinska Institutet Stockholm, Sweden; R. Handels, Alzheimer Centre Limburg, Department of Psychiatry and Neuropsychology, Maastricht University, 6200 MD Maastricht, Netherlands; M.L. Moro, Regione Emilia Romagna, Italy; M.C. Jori, Mediolanum Cardio Research (MCR), Milan, Italy; G. Salvini, Federazione Alzheimer Italia; J. Hugon Center of Cognitive Neurology, APHP, Inserm 1144, University of Paris Lariboisiere Hospital, Paris, France; I. Lazarou, National Observatory for Dementia and Alzheimer's Disease, Health Ministry Athens, Greece; N. Paraskevaidis, Greek Association of Alzheimer's Disease and Related Disorders (GAADRD), Thessaloniki, Macedonia, Hellas.

**RightTimePlaceCare consortium**: The RightTimePlaceCare Consortium partners are: Coordinator: Witten/Herdecke University (DE): Gabriele Meyer, professor (scientific coordinator, WP 1 leader), Astrid Schmitz, Anna Renom Guiteras, Dirk Sauerland, professor (WP 4 & 6 leader), Dr Ansgar Wübker, Patrick Bremer. Consortium Members: Maastricht University (NL): Jan P.H. Hamers, professor (WP 3 leader); Basema Afram, Hanneke Beerens, Dr Michel H.C. Bleijlevens; Dr Hilde Verbeek; Dr Sandra M.G. Zwakhalen. Lund University (SE): Ingalill Rahm Hallberg, professor (WP 2 leader); Ulla Melin Emilsson, professor; Dr. Staffan Karlsson. University of Manchester (UK): David Challis, professor; Caroline Sutcliffe; Dr David Jolley; Anthony Crook; University of Turku (FI): Helena Leino-Kilpi, professor; Jaana Koskenniemi, Riitta Suhonen, professor; Matti Viitanen, professor; Seija Arve, adj professor; Minna Stolt; Dr. Maija Hupli; University of Tartu (EE): Kai Saks, professor (WP 5 leader); Ene-Margit Tiit, professor; Jelena Leibur; Katrin Raamat; Angelika Armolik; Teija Tuula Marjatta Toivari; Fundació Privada Clinic per la Recerca Biomedica, Hospital Clinic of Barcelona (ES): Dr Adelaida Zabalegui (WP 5 leader); Dr Montserrat Navarro; Dr Esther Cabrera (Tecnocampus Mataró). Gerontôpole, University of Toulouse (FR): Dr Maria Soto; Agathe Milhet; Dr Sandrine Sourdet; Sophie Gillette; Bruno Vellas, professor.

## Funding sources

There was no funding for the current study.

## Data specific funding/support

**ICTUS**: The ICTUS study was partially supported by a grant from the European Commission within the 5th framework programme (QLK6-CT-2002-02645) and partially from an unrestricted equal grant from each of Eisai, Janssen, Lundbeck, and Novartis pharmaceutical companies. The pharmaceutical companies had no role in study design, data collection, data analysis, data interpretation. Promotion of the ICTUS study was supported by the University Hospital Centre of Toulouse. The data sharing activity was supported by the “Association Monegasque pour la recherche sur la maladie d'Alzheimer” (AMPA) and the UMR 1027 Unit INSERM – University of Toulouse III.

**PLASA**: The PLASA study was supported by a grant from the French Ministry of Health (PHRC 02-006-01). Promotion of the PLASA study was supported by the University Hospital Centre of Toulouse. The data sharing activity was supported by the “Association Monegasque pour la recherche sur la maladie d'Alzheimer” (AMPA) and the UMR 1027 Unit INSERM – University of Toulouse III.

**RightTimePlaceCare**: RightTimePlaceCare was supported by a grant from the European Commission within the 7th Framework Program (project 242153).

See references to publications describing the data pooled in this study for their details on funding.

## Consent statement

The Swedish Ethical Review Authority provided ethical approval for conducting this study (Dnr 2022-01175-01). Each individual participating study had obtained ethics review for the original data collection and analysis. Each individual participating study had obtained informed consent from their participants.

The study was performed in accordance with the ethical standards as laid down in the 1964 Declaration of Helsinki and its later amendments or comparable ethical standards.

Where data was available from the pooled studies diversity was addressed in terms of age and sex.

## Declaration of competing interest

AS No conflict of interest.

BW – no conflict of interest.

CB received outside this study research grants from ZonMw, Alzheimer Nederland/Dutch Alzheimer Society and Gieskes Strijbis fund. He is board member of the Dutch Young-onset Dementia Knowledge Center and member of the IPA YOD shared interest forum and member INTERDEM and its YOD Taskforce (all unpaid).

CGP: No conflict of interest (IQVIA did not give any authorization or funding for this work – as shared data set was not its property and as it was used for research CGP led before integrating IQVIA).

GS have participated, outside this study, at advisory boards for Eisai and Roche, regarding monoclonal antibody drugs for the treatment of Alzheimer's disease.

LF – received research grants unrelated to this work from Hoffmann-LaRoche. Received case honoraria for clinical trials paid to the institution from Axon Neuroscience, Anavex, Alector, Boehringer Ingelheim, Eisai, Hummingbird, NovoNordisk, Noselab; Consulting fees unrelated to this work from Biogen, BioVie, Eisai, Eli Lilly, FOMF, Araclon/Grifols, Janssen Cilag, Medical Tribune, Medfora, Neurimmune, Noselab, NovoNordisk, Roche, TauRX, Schwabe, StreamUp; Honoraria for Clinical study committees from Avanir/Otsuka, PharmatrophiX, Charité Berlin, Neuroscios, Vivoryon.

LJ received research grants unrelated to this work from Vinnova, Innovative Health Initiative, Forte and Novo Nordisk A/S. Consulting fees unrelated to this work from H. Lundbeck A/S, Janssen, Eli Lilly Inc, and license fees for the RUD instrument.

LS: has no competing interest related to the topic of this paper.

OR: No potential conflict of interest to declare.

RH received outside this study consulting fees in the past 36 months from Lilly Nederland and from the Institute for Medical Technology Assessment (paid to institution).

RK received outside this study research grants from ZonMw, Dutch Alzheimer Society and Gieskes Strijbis fund. He is board member of the International Psychogeriatric Association, member of the IPA YOD shared interest forum and member INTERDEM and its YOD Taskforce and taskforce palliative care (all unpaid).

SH – no conflict of interest.

YP has no conflict of interest.

## Appendix Supplementary materials

Image, application 1
