# Supplementary material for: Informal care for people with dementia in Europe
Source: J Prev Alzheimers Dis. 2025 Jan 1;12(1):100015. doi: 10.1016/j.tjpad.2024.100015 (PMC12183986; doi:10.1016/j.tjpad.2024.100015)
Supplement: Supplementary file 1 [file mmc1.docx]

**SUPPLEMENTARY MATERIAL**

Supplementary material related to manuscript entitled “Informal care for people with dementia in Europe” by Handels et al.

*SUPPLEMENTARY 1: HANDLING MISSING DATA*

The sum scores of the various functional disability scales were rescaled by the maximum possible score based on the available items if some but not more than 20% of the items were missing (except for ICTUS, for which 40% was applied). Informal care ‘number of typical days’ was manually imputed with the median of 25 if number of informal care hours was available (n=94). NPI missing frequency or missing severity were imputed with a median of 2 if the item was missing, but the indicator was positive or only the frequency or severity was present (n=184). Missing data on participant age, sex and living situation, and informal caregiver age and sex at follow-up were carried forward from the last available observation.

As described earlier, part of the data were case-wise deleted from analysis mainly due to drop-out (29% at 1-year FU; excluding RTPC as it lacked 1-year FU). To assess the possible presence of a dropout bias, a logistic regression model was fitted to data drop-out status at the next observation using demographic and clinical characteristics at the current observation as predictor. Results indicated cognitive impairment, functional disability, person with dementia age and cohabiting were significantly associated to drop-out (see **Table S1.1** for details), indicating the presence of selective drop-out.

Partly missing data occurred in 30% missing cohabiting status only, 10% missing ADL status only, and 27% missing (a combination of) other factors. To assess whether partly missing data were conditional on observed demographic and clinical characteristics a logistic regression model was fit to data missingness status using the demographic and clinical characteristics at the same observation as predictor. Results indicated a variety of factors significantly associated to missingness (see **Table S1.2** for details), indicating the presence of selective missing.

Therefore, partly missing data on cognitive impairment category, functional disability category, behavioral symptoms category, cohabiting, informal care hours on basic ADL (log transformed), and informal care hours on instrumental ADL (log transformed) were multiple imputed using chained equations and 10 imputed datasets. An ordered logit model was used for categorized cognitive impairment, functional disability and behavioral symptoms, a logit model for cohabiting, and predictive mean matching for informal care hours. All imputed variables were used as predictors as well as age and sex of the patient and family caregiver. See **Figure S1.1** and **Table S1.3** for details on imputation diagnostics.

Table and figure abbreviations:

- ADL, activities of daily living;
- _cat, category (mild, moderate, severe);
- _cp, concerned person with dementia;
- FAQ, mapped Functional Activities Questionnaire;
- _ic, informal caregiver;
- IC, informal caregiver;
- MSE, Mini-Mental State Examination;
- n, number of observations;
- NPI, Neuropsychiatric Inventory;
- PwD, person with dementia;

*Table S1.1: Detailed results of a logistic regression model fit to data drop-out status (dropout yes/no at next observation) using demographic and clinical characteristics at the current observation as predictor.*

*Table S1.2: Results of 5 logistic regression analyses with dependent variable (columns) being missingness status and independent variables (rows) being cognitive impairment (categorized as mild, moderate or severe, based on MMSE), functional disability (categorized as mild, moderate or severe based on mapped FAQ), behavioral symptoms (categorized as mild, moderate or severe, based on NPI-Q), hours of informal care on both instrumental and basic ADL and cohabiting (informal caregiver living together with person with dementia) presenting odds ratio and significance level (*<0.05; **<0.01).*

| predictor | outcome: missing status |  |  |  |  |
| --- | --- | --- | --- | --- | --- |
|  | cognition | function | behavior | Informal care | cohabit |
| n | 9,433 | 10,781 | 9,439 | 8,683 | 4,537 |
| cognition | n/a | 1.49** | 0.93 | 0.83 | 0.90* |
| function | 3.02** | n/a | 1.20** | 0.65** | 1.34** |
| behavior | 1.36** | 1.01 | n/a | 0.65** | 0.50** |
| age PwD | 0.96** | 1.00 | 1.06** | 1.06** | 1.01** |
| sex PwD | 1.01 | 0.76** | 0.95 | 1.26 | 1.31** |
| age IC | 1.00 | 1.00 | 0.97** | 1.01 | 1.00 |
| sex IC | 0.99 | 1.02 | 1.04 | 1.26 | 1.03 |

*Table S1.3: Absolute difference in correlation coefficient between correlation in original data and correlation in imputed data.*

|  | age PwD | sex PwD | age IC | sex IC | cohabit | cognition | function | behavior | informal care basic ADL | informal care instrumental ADL |
| --- | --- | --- | --- | --- | --- | --- | --- | --- | --- | --- |
| age PwD | n/a |  |  |  |  |  |  |  |  |  |
| sex PwD | 0.01 | n/a |  |  |  |  |  |  |  |  |
| age IC | 0.02 | 0.01 | n/a |  |  |  |  |  |  |  |
| sex IC | 0.01 | 0.01 | 0.01 | n/a |  |  |  |  |  |  |
| cohabit | 0.02 | 0.00 | 0.02 | 0.00 | n/a |  |  |  |  |  |
| cognition | 0.00 | 0.01 | 0.02 | 0.00 | 0.01 | n/a |  |  |  |  |
| function | 0.01 | 0.04 | 0.03 | 0.02 | 0.03 | 0.02 | n/a |  |  |  |
| behavior | 0.01 | 0.01 | 0.00 | 0.01 | 0.00 | 0.01 | 0.01 | n/a |  |  |
| informal care basic ADL | 0.02 | 0.06 | 0.05 | 0.03 | 0.02 | 0.02 | 0.02 | 0.07 | n/a |  |
| informal care instrumental ADL | 0.00 | 0.04 | 0.07 | 0.01 | 0.03 | 0.03 | 0.03 | 0.03 | 0.20 | n/a |

*Figure S1.1: Imputation diagnostics of 10 imputed datasets.*

From left top to right bottom:

A: Histogram with density of log transformed basic activities of daily living for original (i.e., observed non-imputed) data (dashed black line) and for imputed data of each of the multiple imputed dataset (solid blue lines).

B: Same as A for log transformed instrumental activities of daily living.

C: Proportion in each category of cognitive impairment (mild, moderate, severe) in the original (i.e., observed non-imputed) data (marked as 0 on horizontal axis) and for imputed data of each of the multiple imputed datasets (marked as 1 to 10 on horizontal axis).

D: Same as C for category of functional impairment.

E: Same as C for category of behavioral symptoms.

F: Same as C for category of informal caregiver cohabiting with person with dementia.

*SUPPLEMENTARY 3: REGRESSION DIAGNOSTICS AND DETAILS OF THE BASE CASE*

*Table S3.1: Regression details: results of the base case statistical model. Dependent variable is informal care time in hours of instrumental and basic activities of daily living per day.*

Abbreviations: cat, category (mild, moderate, severe); cp, concerned person with dementia; FAQ, Functional Activities Questionnaire; ic, informal caregiver; IC, informal care time in hours; MSE, Mini-Mental State Examination; NPI, Neuropsychiatric Inventory.

*Figure S3.1: Regression diagnostic: histogram (vertical axis: density) of the residuals (horizontal axis: residual defined as observed minus predicted value) from the base case regression analysis. Shapiro–Wilk W test for normal data resulted in p<0.001, indicating residuals were not normally distributed.*

*Figure S3.2: Mean of the predicted values per decile of predicted values (i.e., predicted values cut in 10 deciles after which the mean of each decile was calculated) of informal care hours. Horizontal axis: mean predicted informal care hours within deciles of predicted values. Vertical axis: mean observed informal care hours corresponding to their predicted values. Green line is diagonal (i.e., predicted equal to observed value).*

*Figure S3.3: Mean predicted (horizontal axis) and mean observed (vertical axis) values of informal care hours stratified by symptom categories (i.e., combination of categorized cognitive impairment, functional disability and behavioral symptoms). Circle size reflects the size of the subsample.*

*SUPPLEMENTARY 4: ADDITIONAL ANALYSES*

Abbreviations:

- _cons, constant/intercept;
- _cat, category (mild, moderate, severe);
- _cp, concerned person with dementia;
- FAQ_scaled, total score on mapped Functional Activities Questionnaire (range 0-30);
- ic, informal caregiver;
- IC, informal care time instrumental and basic activities of daily living (hours/day);
- MMSE, total score on Mini-Mental State Examination (range 0-30);
- MSE, Mini-Mental State Examination categorized (reference category is mild);
- NPIQ_total_adjusted, total score on Neuropsychiatric inventory (range 0-36);
- Region, reference category is Northern Europe;
- SEX, female sex (reference category is male sex);

*Table S4.1: MMSE categorized; n = 13,529 observations; imputed data.*

*Table S4.2: MMSE total score; n = 12,330 observations; non-imputed data on MMSE total score.*

*Table S4.3: Cognitive impairment, functional disability and behavioral symptoms continuous outcomes; n =* *8,680 observations; non-imputed data on MMSE, mapped FAQ and NPI sum scores.*

*Table S4.4: CDR global score; n =* *4,227 observations; only data from Actifcare and ICTUS studies; non-imputed data on CDR-global (scores 1-3 only, omitting CDR score 0 and 0.5).*

*Table S4.5: CDR sum of boxes categorized; n = 4,399 observations; only data from Actifcare and ICTUS studies; non-imputed data on CDR sum of boxes (categorized from scores 4.5-18 only).*

*Table S4.6: CDR sum of boxes; n =* *5,791 observations; only data from Actifcare and ICTUS studies; non-imputed data on CDR sum of boxes (scores 0-18).*

*Table S4.7: Additional data from Gervès-Pinquié et al. [*[*https://doi.org/10.1016/j.healthpol.2014.01.001*](https://doi.org/10.1016/j.healthpol.2014.01.001)*] and Pires et al. [*[*https://doi.org/10.20344/amp.11922*](https://doi.org/10.20344/amp.11922)*] with MMSE or if missing Global Deterioration Scale (GDS); n = 14,006 observations; adjusted for person with dementia age and sex only; non-imputed data on GDS (scores 4-7 only).*

*The study by Gervès-Pinquié et al. [*[*https://doi.org/10.1016/j.healthpol.2014.01.001*](https://doi.org/10.1016/j.healthpol.2014.01.001)*] had the following characteristics. Main aim: assess costs of community-dwelling patients with AD and factors associated to informal care. Design: cohort. Sample size received data: 58. Planned observations at months: 0 (baseline only). Recruitment settings: regional public multi-disciplinary memory clinics located in mid-sized towns referred by GPs. Countries: France. Cognition: MMSE. Informal care: RUD.*

*The study by Pires et al. [*[*https://doi.org/10.20344/amp.11922*](https://doi.org/10.20344/amp.11922)*] and Paúl et al. [*[*https://doi.org/10.1007/s10597-018-0345-6*](https://doi.org/10.1007/s10597-018-0345-6)*] had the following characteristics. Main aim: quantify health and social services use and caregiving time in dementia. Design: cohort. Sample size received data: 123. Planned observations at months: 0 (baseline only). Recruitment settings: community partners such as primary health care centers. Countries: Portugal. Cognition: Global Deterioration Scale (GDS). Informal care: RUD.*

*SUPPLEMENTARY 5: SENSITIVITY ANALYSES*

*Figure S5.1: Regression model beta coefficients for cognitive impairment, functional disability and behavioral symptoms categorized as moderate or severe (mild is reference category) from first and second sensitivity analyses.*

*Sensitivity analysis 1a ‘sum IC before imputation’: hours of instrumental and basic ADL were summed before imputation, then the base case statistical model was applied. Sample size was smaller because in 278 (2%) observations informal care could not be summed due to either instrumental or basic ADL informal care hours missing.*

*Sensitivity analysis 1b ‘add separate IC domains’: base case statistical model applied separately to instrumental ADL and basic ADL (i.e., two separate regression models). Coefficients of cognitive impairment, functional disability and behavioral symptoms for instrumental ADL and basic ADL domains were summed manually to reflect their combined impact.*

*Sensitivity analysis 2a-d: the base case statistical model and a selection of the statistical models among the additional analyses were applied to the same subsample of 4,185 observations that had CDR global, CDR sum of boxes and categorized cognitive impairment, functional disability and behavioral symptoms data available, being Actifcare and ICTUS studies only.*

*Figure S5.2: Base case statistical model beta coefficients for cognitive impairment, functional disability and behavioral symptoms categorized as moderate or severe (mild is reference category) from each specific study.*
